# Automatic Recognition of Sucker-Rod Pumping System Working Conditions Using Dynamometer Cards with Transfer Learning and SVM

**DOI:** 10.3390/s20195659

**Published:** 2020-10-03

**Authors:** Haibo Cheng, Haibin Yu, Peng Zeng, Evgeny Osipov, Shichao Li, Valeriy Vyatkin

**Affiliations:** 1 State Key Laboratory of Robotics, Shenyang Institute of Automation, Chinese Academy of Sciences, Shenyang 110016, China; chenghaibo@sia.cn (H.C.); zp@sia.cn (P.Z.); lishichao@sia.cn (S.L.); 2 Key Laboratory of Networked Control Systems, Chinese Academy of Sciences, Shenyang 110016, China; 3 Institutes for Robotics and Intelligent Manufacturing, Chinese Academy of Sciences, Shenyang 110169, China; 4 University of Chinese Academy of Sciences, Beijing 100049, China; 5 Department of Computer Science, Electrical and Space Engineering, Luleå University of Technology, 97187 Luleå, Sweden; evgeny.osipov@ltu.se (E.O.); valeriy.vyatkin@ltu.se (V.V.); 6 Department of Electrical Engineering and Automation, Aalto University, 02150 Espoo, Finland

**Keywords:** working condition recognition, sucker-rod pumping system, dynamometer card, convolutional neural network, transfer learning, support vector machine

## Abstract

Sucker-rod pumping systems are the most widely applied artificial lift equipment in the oil and gas industry. Accurate and intelligent working condition recognition of pumping systems imposes major impacts on oilfield production benefits and efficiency. The shape of dynamometer card reflects the working conditions of sucker-rod pumping systems, and different conditions can be indicated by their typical card characteristics. In traditional identification methods, however, features are manually extracted based on specialist experience and domain knowledge. In this paper, an automatic fault diagnosis method is proposed to recognize the working conditions of sucker-rod pumping systems with massive dynamometer card data collected by sensors. Firstly, AlexNet-based transfer learning is adopted to automatically extract representative features from various dynamometer cards. Secondly, with the extracted features, error-correcting output codes model-based SVM is designed to identify the working conditions and improve the fault diagnosis accuracy and efficiency. The proposed AlexNet-SVM algorithm is validated against a real dataset from an oilfield. The results reveal that the proposed method reduces the need for human labor and improves the recognition accuracy.

## 1. Introduction

The significance of oil as a global energy source is difficult to overemphasize. The increased demand for oil is forcing petroleum engineers to determine new strategies to increase oil production. To exploit oil efficiently and rapidly from underground reservoirs, researchers have studied artificial lift methods. The sucker-rod pumping system, as the most widely applied type of artificial lift method for production wells, has been commonly adopted in the oil and gas industry.

**Figure 1:**
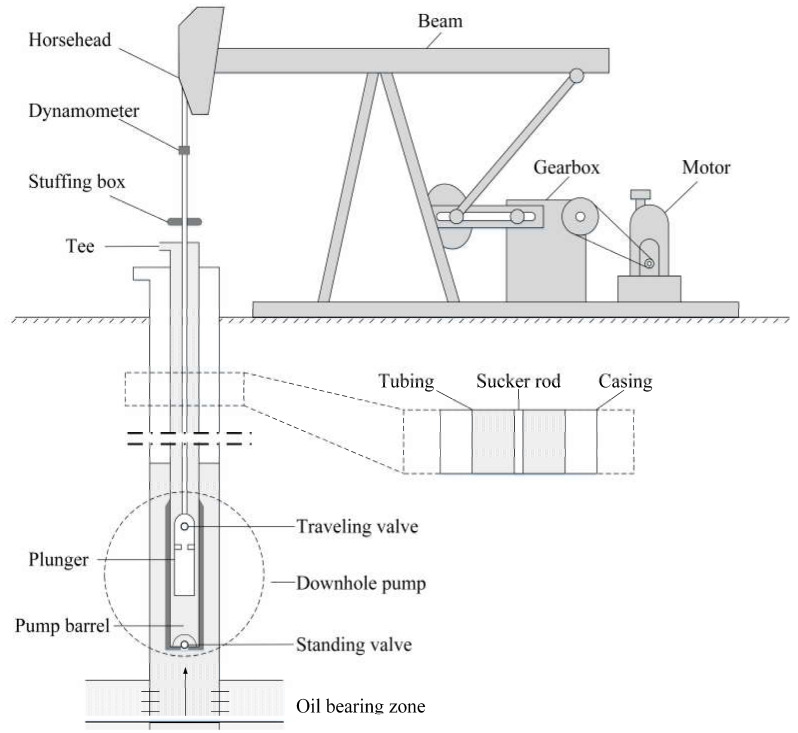
Schematic of a typical production unit with the major components of the sucker-rod pumping system. Dynamometer is installed on the rod of the pumping unit, and used to measure load on the polished rod and plot the load in relation to the rod displacement as the pumping unit moves through a stroke cycle.

The sucker-rod pumping system is composed of several components, some of which operate aboveground, while other parts operate underground, i.e., down the well. The basic and primary elements include the prime mover, pumping unit, sucker rod, and downhole pump. Figure 1 shows a detailed schematic of a typical production unit with the major components of the sucker-rod pumping system.

Thousands of the aforementioned production units occur in oilfields, which are distributed spatially across the whole field, and operate for decades in the field. Therefore, it is difficult to monitor and manage these production units. Moreover, many unexpected exceptions may occur in the sucker-rod pumping system during long-term operation due to the complexity of the production environment. The issue is how to automatically recognize and diagnose the system working conditions in real time. Currently, this work is mainly performed by manually analyzing the shape of dynamometer cards.

Dynamometer card diagnosis is the most widely adopted method to evaluate the working conditions of the sucker-rod pumping system. The dynamometer card is a closed curve that represents the relationship between the displacement and load of the sucker-rod pump. The card shape generally reflects the condition of the pumping well, and different conditions can be indicated by their typical characteristics on the cards [1,2,3]. Thus, the manual recognition and diagnosis of working conditions are a time-consuming and labor-intensive task, and this process usually requires specialized domain knowledge. Moreover, the massive data collected by sensors, such as dynamometer cards, temperature, and pressure data, are more difficult for domain experts to process. The accurate and efficient identification and diagnosis of the working conditions of sucker-rod pumping wells by utilizing oilfield big data are a new challenge for researchers.

With the rapid development of smart manufacturing and artificial intelligence technologies, several articles have attempted to review and summarize the intelligent methods for machine abnormalities and faults diagnosis. Jiao et al. [4] reviewed convolutional neural network based machine fault diagnosis approaches. A typical convolutional network based fault diagnosis framework was proposed. It is composed of data collection, model construction, and feature learning and decision making. Gao et al. [5,6] summarized four different fault diagnosis methods, which are model-based, signal-based, knowledge-based and hybrid/active approaches, respectively. These papers also introduced the advantages and constraints of each techniques. In [7], fault diagnosis and remaining useful life estimation were presented to deal with these problems. Liu et al. [8] presented a comprehensive review of artificial intelligence algorithms in rotating machinery fault diagnosis, from both the views of theory background and industrial applications. Zhao et al. [9] summarized four kinds of deep learning methods and their applications to machine health monitoring. Lei et al. [10] presented a review of condition monitoring and fault diagnosis of planetary gearboxes. Hoang et al. [11] presented a survey on deep learning based bearing fault diagnosis, in which three deep learning methods and their applications were summarized.

In this paper, a dynamometer card based automatic fault diagnosis method is proposed to classify the working conditions of sucker-rod pumping systems. Two steps are involved in this method. Firstly, AlexNet-based transfer learning is applied to automatically extract representative features from various dynamometer cards. Secondly, with these extracted features, an error-correcting output codes (ECOC) model-based SVM is designed to identify the working conditions and improve the fault diagnosis accuracy and efficiency.

This paper is organized as follows: Section 2 introduces the related works on dynamometer card-based intelligent fault diagnosis methods for sucker-rod pumping systems. The fault diagnosis problem is stated in Section 3. Section 4 details the proposed hybrid AlexNet-SVM method. In Section 5, the diagnosis results obtained with the proposed method are discussed. Finally, conclusions and future works are provided in Section 6.

## 2. Related Works

**Figure 2:**
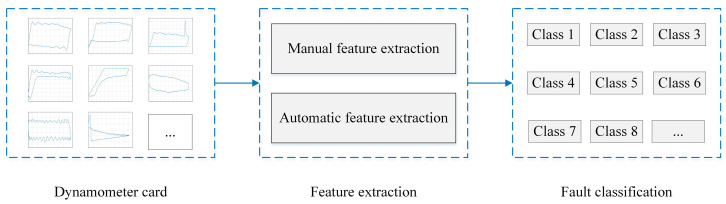
The process of intelligent fault diagnosis for suck-rod pumping systems.

Dynamometer card-based intelligent fault diagnosis of sucker-rod pumping systems is a new and challenging topic in the oil and gas industry. Over the last few decades, many advanced diagnosis methods have been proposed to analyze the downhole working conditions of production wells. Generally, feature extraction and fault classification are two major processes for the intelligent identification and diagnosis of suck-rod pumping systems, as shown in Figure 2. According to the feature extraction method, the fault diagnosis problem can be divided into two categories: machine learning-based methods with manual feature extraction and deep learning based methods with automatic feature extraction.

### 2.1. Machine Learning-Based Fault Diagnosis Methods with Manual Feature Extraction

Expert systems have been applied to diagnose the working conditions of sucker-rod pumping systems [12,13,14,15], and pattern recognition was first introduced into the fault diagnosis approach of dynamometer cards [15]. Expert system and pattern recognition could effectively reduce expertise required for fault diagnosis of pumping wells with considerable savings in labor hours. However, the accuracy of expert systems greatly depends on the domain knowledge and experience of experts.

With the rapid development of artificial intelligence techniques, neural networks have been widely applied in the intelligent fault recognition of dynamometer cards [16,17]. The application of neural networks in pattern recognition requires a large number of training samples to ensure the accuracy of the recognition effect. In oilfields, the pumping system needs to be immediately shut down under certain conditions, especially serious faults, such as sucker-rod breakage. Therefore, the number of samples corresponding to these cases is limited, and it is difficult to meet the training requirements of neural networks.

SVM, as a powerful and flexible supervised machine learning algorithm, has been adopted to identify the working conditions of sucker-rod pumping wells [2,18,19], and they can effectively solve the problem of constructing high-dimensional data models with limited samples. However, it is difficult to use traditional SVMs to resolve multiple classification problems. Moreover, the representative features of the dynamometer cards must be extracted before fault classification. The feature extraction process is primarily based on mechanism analysis, prior knowledge, and expert experience, which means the accuracy is greatly influenced by external factors, and cannot be strictly guaranteed.

During manual feature extraction, high-dimensional dynamometer card data are transformed into a series of low-dimensional features by utilizing the above machine learning methods. Some valid and useful information will inevitably be lost. Therefore, certain working conditions cannot be accurately or correctly identified. In addition, this process largely depends on diagnostic expertise and prior knowledge, and it is time-consuming and labor-intensive [20]. The challenge is how to extract the features automatically and identify the faults accurately for sucker-rod pumping system in oilfield.

### 2.2. Deep Learning-Based Fault Diagnosis Methods with Automatic Feature Extraction

To solve the problem of information loss caused by manual feature extraction, deep learning-based fault diagnosis methods with automatic feature extraction have been proposed over the past few years. Deep learning-based methods automatically extract image features from dynamometer cards by utilizing advanced deep neural networks. Convolutional neural networks (CNNs), as artificial deep learning neural networks, have been applied for fault diagnosis of sucker-rod pumping systems in recent years.

Zhao et al. [1] proposed data-based CNN and image-based CNN methods for fault diagnosis of rod pumping system, and compared the proposed methods with traditional machine learning algorithms. The results demonstrated that CNN-based approach is superior to the conventional approaches without any need of manual feature extraction that requires domain expertise. In [21], the potential of using artificial neural networks in well fault diagnosis was reviewed and analyzed. VGG16, ResNet34, and ResNeXt50 were used to recognize beam pump conditions based on a pump card shape. In [22], a fourteen-layer CNN diagnosis model was proposed recognize working conditions of sucker rod pumping wells based on big data deep learning. In [23], Peng applied artificial intelligence in sucker rod pumping wells. Deep neural networks based method was used to realize intelligent dynamometer card generation, diagnosis, and failure detection. In this method, CNN and autoencoders were adopted to get the feature representation of the dynamometer cards and classify the working conditions of sucker rod pumping wells. The accuracy and efficiency have greatly been improved by these approaches. However, the conducted studies using deep learning have mostly relied on neural networks trained from scratch, which generally requires numerous epochs or iterations for a deep neural network to converge [21]. Some methods need an amount of time to train the model and classify the pattern. Therefore, they cannot meet the real-time requirement of industrial application.

## 3. Problem Statement

**Figure 3:**
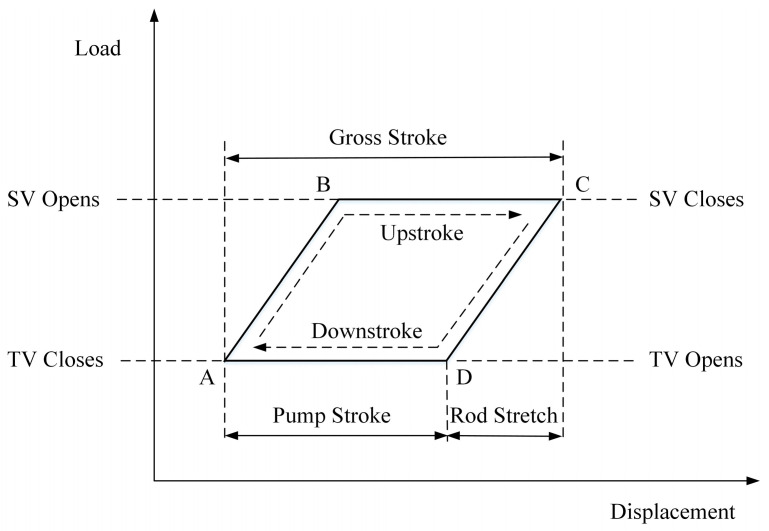
Theoretical dynamometer card under static load [23].

The horsehead equipment, sucker rod and down hole pump are the major and closely related components involved in the production process of oil and gas, as shown in Figure 1. With the rod moving up and down, the traveling valve (TV) attached to the rod and the standing valve (SV) at the bottom of the pump open and close periodically, which drives the oil and gas to the surface from the underground reservoir [23]. The journey of the rod traveling from the upper dead point to the lower dead point and back up again is called a stroke. The dynamometer card displays the load on the sucker rod over a stroke. This is the major approach to evaluate and diagnose the working conditions of sucker-rod pumping systems. The shape of the card indicates the working conditions and performance of the pump. Figure 3 shows a theoretical card under a static load.

In this paper, the 7 most commonly known fault conditions are investigated. Therefore, there are 8 classification categories in this research, in which the normal operation condition (NOC) is regarded as a separate class. These categories are NOC, downstroke pump bumping (DPB), upstroke pump bumping (UPB), combination of leaking standing and traveling valves (CST), gas interference (GIF), insufficient liquid supply (ILS), sand production (SAP), and abnormal dynamometer card (ADC). The ADC category indicates all the abnormal shapes of the dynamometer card caused by the data losses and errors in the sensor sampling and transmission processes.

**Figure 4:**
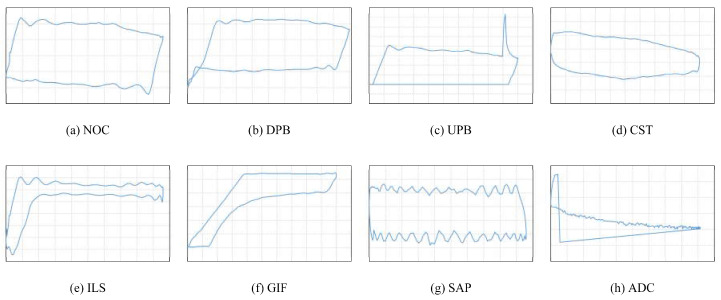
Typical working conditions of the sucker-rod pumping system, where the horizontal axis denotes the displacement, and the vertical axis denotes the load: (**a**) normal operation condition, (**b**) downstroke pump bumping, (**c**) upstroke pump bumping, (**d**) combination of leaking standing and traveling valves, (**e**) insufficient liquid supply, (**f**) gas interference, (**g**) sand production, (**h**) abnormal dynamometer card.

Figure 4 shows the typical working conditions of sucker-rod pumping systems, where the horizontal axis denotes the displacement, and the vertical axis denotes the load. As shown in this figure, each of these categories exhibits specific features reflected in the dynamometer card shape. Figure 4h shows one of the ADC possibilities.

## 4. Methodology

In this paper, a hybrid AlexNet-SVM-based model is proposed to diagnose the various fault categories of sucker-rod pumping systems. AlexNet-based transfer learning is applied to automatically extract useful and representative features from dynamometer cards. With the extracted features, an ECOC model-based SVM is designed to classify the working conditions of the pumping system and improve the pattern recognition efficiency.

### 4.1. Convolutional Neural Networks and Transfer Learning

CNNs are typical feedforward neural networks with convolutional computations and deep structures. CNNs, as some of the most representative deep learning models, have been widely applied in many fields, and numerous related applications, including image classification [24,25,26,27,28], natural language processing [29,30], face recognition [31,32], video analysis [33,34], and pedestrian detection [35,36].

**Figure 5:**
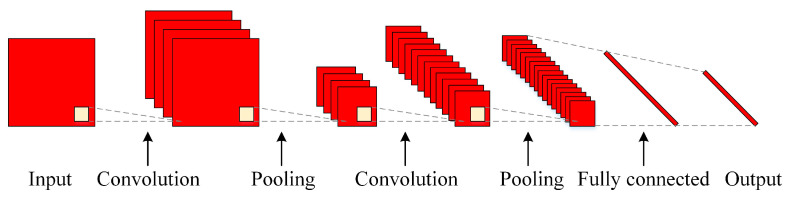
Typical CNN architecture.

A typical CNN architecture is shown in Figure 5. It consists of an input layer, an output layer, and multiple hidden layers. The hidden layers are composed of a series of convolutional layers (Conv), pooling layers, and fully connected layers (FC). The Conv layer is the key functional block of a CNN, which convolutes a filter matrix with values from a receptive field of neurons [37] and finally extracts representative features from input data.

Transfer learning imitates the human visual system by taking full advantage of prior knowledge in different but related domains when executing new tasks in a given domain and resolves relevant cross-domain learning problems [38]. In transfer learning, representative information is extracted from data in the related domain by a pretrained model, and the pretrained model then transfers the useful information for reuse on a new target problem. Generally, transfer learning provides three kinds of benefits for performance improvements [38,39,40], including (1) a higher start with an improved performance at the initial points; (2) a higher slope with a faster performance growth; and (3) a higher asymptote, producing a better final performance. Deep learning generally requires a large amount of data to train deep neural networks and learn the knowledge [41]. However, transfer learning trains networks with comparatively little data because of the pretrained model. This is very significant since most real-world problems and tasks typically do not have millions of labeled data to train such complex models.

### 4.2. Support Vector Machine

The SVM is a successful and important supervised machine learning method used to address classification problems on the basis of the principles of empirical risk minimization and structural risk minimization [42]. SVMs have been widely applied in many areas [43,44]. The basic idea of the SVM is to seek the optimal hyperplane in the feature or sample space under the maximum margin principle.

The SVM was initially proposed to solve two-class problems. Given a sample dataset including N points ${\left\{x_{k},y_{k}\right\}}_{k=1}^{N}$, where $x_{k}\in R^{n}$ are the input data, and y∈{±1} is the target output, the optimal hyperplane is defined as:

(1) $$\omega^{T}x_{k}+b=0$$

where ω is the weight vector and *b* is the bias term. The parameters ω and *b* are determined as:

(2) $$y_{k}\left(\omega^{T}x_{k}+b\right)\geq 1-\xi_{k}$$

where $\xi_{k}$ is the slack variable, and $\xi_{k}\geq 0$.

(3) $$\Phi \left(\omega ,\xi \right)=\frac{1}{2}{\left\|\omega \right\|}^{2}+C\sum_{k=1}^{N}\xi_{k}$$

where *C* is a penalty coefficient, and C≥0. Considering the Lagrangian multiplier method, the solution to the optimal hyperplane can be determined as:

(4) $$Q\left(\lambda \right)=\sum_{i=1}^{N}\lambda_{i}-\frac{1}{2}\sum_{i=1}^{N}\sum_{j=1}^{N}\lambda_{i}\lambda_{j}y_{i}y_{j}K(x_{i},x_{j})$$

where $\lambda_{i}$ is the Lagrange multiplier and $K(x_{i},x_{j})$ is the kernel function. Kernel functions are designed to solve the inner product operation in high-dimensional space, thus addressing the problem of nonlinear classification. These kernel functions can be of different types, such as linear, polynomial, Sigmoid, hyperbolic tangent and radial basis function.

### 4.3. Proposed AlexNet-SVM Method

**Figure 6:**
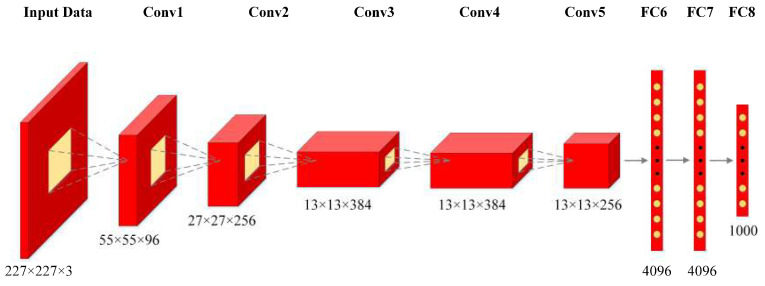
The main layers of AlexNet.

In this study, an AlexNet-based CNN network is proposed to automatically extract the representative features from various dynamometer cards. AlexNet, as a well-known and successful deep CNN for image classification, attained the highest accuracy during the ImageNet Large Scale Visual Recognition Challenge in 2012 [24]. It is mainly composed of eight layers, including five Conv layers and three FC layers, as depicted in Figure 6. 

Considering training time with gradient descent, the saturating nonlinearities are much slower than the non-saturating nonlinearity *f*(*x*) = max (0, *x*) [24]. In AlexNet, therefore, the Rectified Linear Units layer (ReLU) layer is adopted as the activation function layer after every main layer, except the last FC layer, to improve the training time and learning performance of the neural network. Normalization layers follow the first two Conv layers. Max-pooling layers follow the normalization layers as well as the fifth Conv layer. They are adopted to downsample and reduce the size of the neural network. To reduce the overfitting degree in the FC layers, dropout layers are designed after the first two FC layers. After the last FC layer, a Softmax layer is applied to produce the distribution over the input data.

The SVM is a widely applied classification method to solve two-class problems. However, the working condition recognition problem of sucker-rod pumping systems is a multiclass problem. Thus, an ECOC model-based multiclass SVM is proposed. The ECOC approach is a meta-method that combines many binary classifiers. To solve the multiclass problem, it reduces the classification problem with three or more classes to a set of binary classification problems. In the ECOC model, the error caused by poor choices of input features, finite training data, and flaws in the training algorithms can be reduced by employing redundant error-correcting bits [45]. The ECOC model requires a coding design, which determines the classes that the binary learners are trained on, and a decoding scheme, which determines how the results of the binary classifiers are aggregated. The common coding designs include one-versus-all, one-versus-one, binary complete, ternary complete, ordinal, dense random, and sparse random designs.

**Figure 7:**
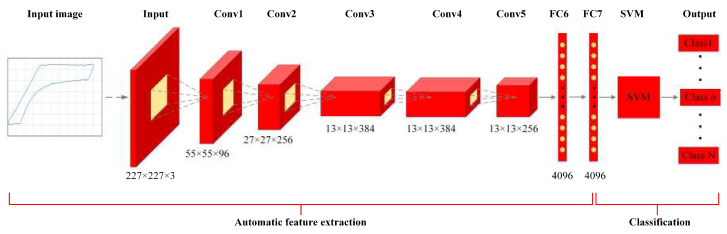
Architecture of the proposed AlexNet-SVM method. This method consists of automatic feature extraction and classification processes. For feature extraction, AlexNet-based transfer learning automatically extracts the useful and representative features from various dynamometer cards. With these extracted features, the ECOC model-based SVM is designed to classify the working conditions of the pumping systems.

To combine the advantages of these two methods, a hybrid AlexNet-SVM method is proposed in this paper, as shown in Figure 7. The proposed method is an image-based algorithm. It takes as input the dynamometer cards collected from oilfield. The input images need to be adjusted from the original size to 227×227×3 to accommodate the input pixel requirement of AlexNet. This algorithm consists of automatic feature extraction and fault classification processes. For feature extraction, the seven-layer AlexNet-based neural network is adopted to automatically extract useful and representative features from dynamometer cards. Five Conv and two FC layers are involved in this process. The Conv1 layer filters the 227×227×3 input image with 96 kernels of size 11×11×3. The Conv2 layer filters the output of Conv1 layer with 256 kernels of size 5×5×48. The number of kernels for Conv3, Conv4, and Conv5 layers are 384, 384, and 256, and the sizes of corresponding kernels are 3×3×256, 3×3×129, and 3×3×129, respectively. The FC6 and FC7 layers have 4096 neurons each. For fault classification, ECOC model-based SVM is adopted to identify the faults of sucker-rod pumping system. SVM takes as input the output of the FC7 layer. Using this method, the network classifies and outputs the estimated working conditions of the pumping system.

The AlexNet-based network is trained first on the base dataset and target, and then the learned features are transferred to proposed method to realize working condition recognition target based on our dataset. With transfer learning approach, the first seven layers are copied to the first seven layers of our network. The SVM parameters are randomly initialized and trained toward the target task. However, the parameters and features are fine-tuned to the new task to improve performance. In this process, AlexNet-based transfer learning automatically extracts the useful and representative features from various dynamometer cards. With these extracted features, the ECOC model-based SVM is designed to classify the working conditions of the pumping systems and improve the fault diagnosis accuracy and efficiency.

**Figure 8:**
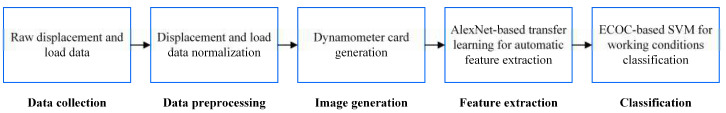
Workflow of the methodology proposed for working conditions recognition of pumping system.

The organization and schematic diagram of the methodology proposed in this study for working condition recognition of pumping systems is depicted in Figure 8. This process is categorized into five parts: data acquisition, data preprocessing, dynamometer card generation, feature extraction, and working condition classification.

- Raw displacement and load data are collected by card collection sensors, called dynamometer, during daily operations in an oilfield. A dynamometer is a valuable device used on sucker-rod pumps that measures load on the polished rod and plots the load in relation to the rod displacement as the pumping unit moves through a stroke cycle. Dynamometer data can be used to select equipment, recognize operating conditions, and reduce trouble of installed equipment.
- The collected data are then preprocessed and normalized to the range of [0,1] to eliminate any mutual effects between the extremely large and small values in the dataset. The common data normalization methods include Z-score, log scaling, and Min-Max normalization.
- Dynamometer card is generated based on the normalized load and displacement data. As seen in Figure 3, the horizontal axis is the displacement of the sucker-rod pumping system, and the vertical axis is the load. Dynamometer card is a plot of load versus displacement on the rod. It is useful for surveillance purposes. The card shape reflects the operating condition of the pumping well, and different conditions can be indicated by their typical characteristics on the cards.
- The AlexNet-based transfer learning technique is applied to extract the useful and representative features from the generated dynamometer cards. With this method, the gained knowledge from pretrained neural network can be applied to a different but related problem, such as the working condition recognition of sucker-rod pumping system in this study. The neural network needs not train from scratch, which speeds up the training process of the network.
- Finally, the working conditions of the pumping system are classified by the ECOC-based SVM method.

## 5. Experiment and Results

This section describes the experiment conducted and the results obtained in terms of working condition recognition of the pumping system using AlexNet-based transfer learning and ECOC-based SVM.

**Figure 9:**
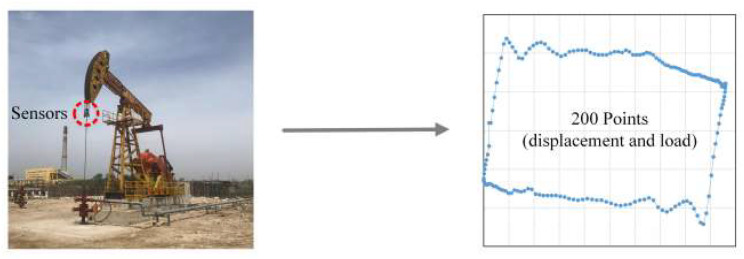
Data collection of sucker-rod pumping system. These sensors are dynamometers. They are used to collect displacement and load data. Based on these data, the dynamometer cards can be generated, where the horizontal axis is the displacement of the sucker-rod pumping system, and the vertical axis is the load.

All the data used in this experiment were collected from a real oilfield in northern China, and the data were measured by sensors attached to sucker rods. These sensors are dynamometers installed on the rod. They are small in size and light in mass. Therefore, they can be installed on the equipment to be measured in a very simple and convenient. In oilfield, dynamometers are used to measure load on the polished rod and displacement of the pumping unit. Then the collected data is sent to data center via wireless network. Based on the displacement and load data, dynamometer cards were generated, as shown in Figure 9.

In this study, 8 different working conditions are considered, including NOC, DPB, UPB, CST, GIF, ILS, SAP, and ADC (refer to Section III). For each working condition, 1000 samples are selected for recognition and classification purposes, and all the samples are randomly divided into training and testing datasets. Eighty percent of the total dataset is adopted to train the proposed model, and the remaining 20% is adopted to test the model. To accelerate the training process, an NVIDIA GeForce MX150 with MATLAB is employed in this experiment. 

The displacement (*D*) and load (*L*) data are collected from different wells by different sensors in the oilfield. To remove discrepancies between the different wells and sensors, the acquired data are normalized using the Min-Max normalization method. The normalization method can be described as:

(5) $$D_{i}^{*}=\frac{D_{i}-\min(D)}{\max(D)-\min(D)}$$

(6) $$L_{i}^{*}=\frac{L_{i}-\min(L)}{\max(L)-\min(L)}$$

where $D_{i}^{*}$ and $L_{i}^{*}$ are the normalized displacement and load, respectively, for i=1,2,…,200, and max(*D*), max(*L*), min(*D*) and min(*L*) are the maximum displacement, maximum load, minimum displacement and minimum load, respectively.

After the displacement and load data are normalized to the range of [0,1], the data can be transformed into various dynamometer cards. To implement AlexNet-based transfer learning method, the input images are adjusted from the original size to 227×227 to accommodate the input pixel requirement of AlexNet. As shown in Figure 7, the extracted features from FC7 of AlexNet are selected as the input of the SVM classifier, which is a compromise between the classification accuracy and computational complexity.

For the multiclass SVM, the linear kernel function is adopted to accelerate the training process. Moreover, linear kernel function is less prone to overfitting than non-linear functions. The one-versus-one code design is employed to design the ECOC, and the code length can be determined as:

(7) $$\frac{K\times (K-1)}{2}$$

where *K* is the number of classes. Hence, each code has a length of 28 in this study, and the coding design is provided in Table 1.

To evaluate the performance and efficiency of the proposed AlexNet-SVM method for working condition recognition, the classification results of the proposed method are compared to those of the classical AlexNet algorithm, in which Softmax is applied in the output layer to classify the image input, VGG16 and ResNet34. The overall classification accuracy rate is defined by the total number of correctly classified samples divided by the total number of all samples. The overall accuracy rate is summarized in Table 2. The overall classification accuracy of the proposed AlexNet-SVM method is higher than 99%. This is of great importance considering the diversity and complexity of the dynamometer cards generated by the massive sensor data. As can be seen from Table 2, the best method is AlexNet-SVM, and the overall classification accuracy is 99.50%. For other methods, the classification accuracy increases along with the depth of the CNN networks, such as eight-layer AlexNet, sixteen-layer VGG16, and thirty-four-layer ResNet34. In this case, SVM-based classification method achieves better accuracy than FC-based method within a given time step.

**Figure 10:**
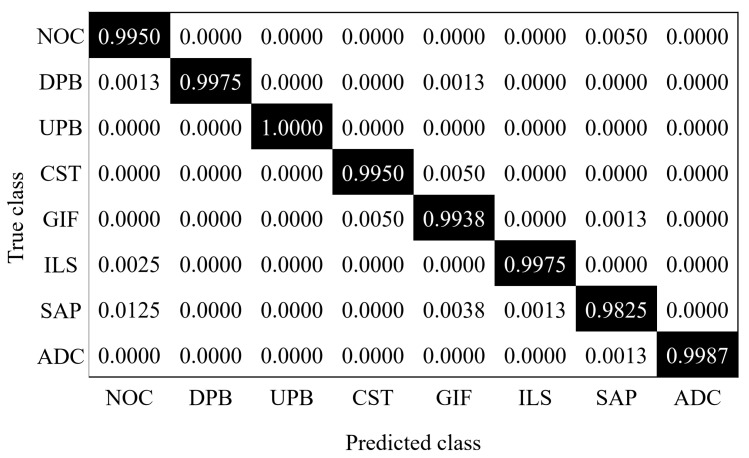
Confusion matrix of the proposed method on dynamometer card dataset.

In industrial applications, real-time capability is a significant performance index. Using this method, four photos can be identified per second, which means that the proposed method meets the real-time requirement. To show more details about the working conditions recognition of sucker-rod pumping system, the confusion matrix of the proposed AlexNet-SVM method is given in Figure 10. The ratios on the diagonal of the confusion matrix are the proportions of the samples that were correctly classified in each operating condition, and the off-diagonal are the misclassified samples proportion. As shown in this figure, most of the ratios on the diagonal are greater than 0.99, especially for class NOC, DPB, UPB, CST, ILS, and ADC, which shows that most of samples are correctly classified. For UPB condition, it can be exactly recognized based on these samples. However, the proposed method misclassifies 1.25% of samples of the SAP as the NOC and 0.5% of samples of the NOC as the SAP. The reason may be that the shapes of the NOC and the SAP conditions are irregular quadrilateral. The results are accordance with the fact that there is only small difference between some of NOC and SAP dynamometer cards, which makes it more difficult to distinguish the two working conditions than other conditions. Most of classes are only misclassified from one class, such as NOC, CST, ILS and ADC. Whereas, Some other conditions are most likely to be misclassified as conditions SAP, including NOC, GIF, and ILS.

To analyze the effect of the network structure and extracted features on the classification accuracy, the outputs of layers FC6 and FC8 of AlexNet are selected as the input of the SVM classifier. The results are compared to the proposed AlexNet-SVM method, in which the input of SVM is the output of layer FC7 (as shown in Figure 7), and the accuracy rates are summarized in Table 3. The structures of FC6-SVM and FC7-SVM attain higher accuracy rates than that of FC8-SVM because the first two structures exhibit a more abstract feature input, while the FC8 layer skews more towards concrete classes.

As for computation complexity and time cost, CNN generally needs more training time than traditional machine learning methods due to a huge number of weight parameters to be trained, especially when the CNN network has a deeper structure. Besides, more computing resources, such as hardware configuration, are needed for a faster training process. Some compromises need to be made before training a deep learning network. However, as the fast development of hardware, such as graphic processing units, deep learning-based methods are becoming more reliable and efficient for industrial applications.

## 6. Conclusions and Future Work

Pumping wells are widely distributed across oilfields, and operate over decades of these fields. Thus, it is difficult to monitor and recognize the working conditions of these production units. Moreover, certain unexpected exceptions may occur in the sucker-rod pumping system during long-term operation due to the complexity of the production environment. The traditional manual recognition methods are time-consuming and labor-intensive. Therefore, an automatic fault diagnosis method is proposed to recognize the working conditions of sucker-rod pumping systems with massive dynamometer card data collected by sensors. In this method, AlexNet-based transfer learning is implemented to automatically extract the useful and representative features from various dynamometer cards. With these extracted features, an ECOC model-based SVM is designed to classify the working conditions and improve the fault diagnosis accuracy. The overall classification accuracy exceeds 99%, which demonstrates that the proposed AlexNet-SVM method is an efficient method for working condition recognition of sucker-rod pumping systems. In addition, three different network structures are compared to analyze the effect of the network structure and extracted features on the classification accuracy. The proposed method could be generalized to all possible working conditions of sucker-rod pumping systems as long as relevant dataset is well provided. This is the first time to combine deep learning and traditional machine learning method in this problem, which can effectively reduce the need for human labor, and improve the recognition accuracy.

In future work, we plan to collect more data on special working conditions to monitor and recognize more types of working conditions of pumping wells. Various sensor data, such as power, current, and temperature data, will be considered to improve the classification accuracy and algorithm performance. Moreover, it would be noteworthy to study the dynamic changes in working conditions of sucker-rod pumping systems in a timely and accurate manner with various sensor data.

## Figures and Tables

**Table 1:** A 28-bit ECOC for the eight-class working condition recognition problem.

Class		0	1	2	3	4	5	6	7
Code Word	f_0_	1	−1	0	0	0	0	0	0
	f_1_	1	0	−1	0	0	0	0	0
	f_2_	1	0	0	−1	0	0	0	0
	f_3_	1	0	0	0	−1	0	0	0
	f_4_	1	0	0	0	0	−1	0	0
	f_5_	1	0	0	0	0	0	−1	0
	f_6_	1	0	0	0	0	0	0	−1
	f_7_	0	1	−1	0	0	0	0	0
	…	…	…	…	…	…	…	…	…
	f_20_	0	0	0	1	0	−1	0	0
	f_21_	0	0	0	1	0	0	−1	0
	f_22_	0	0	0	1	0	0	0	−1
	f_23_	0	0	0	0	1	−1	0	0
	f_24_	0	0	0	0	1	0	−1	0
	f_25_	0	0	0	0	1	0	0	−1
	f_26_	0	0	0	0	0	1	−1	0
	f_27_	0	0	0	0	0	1	0	−1

**Table 2:** Overall classification accuracy of the proposed method.

Method	AlexNet	VGG16	ResNet34	AlexNet-SVM
Accuracy	95.64%	96.48%	97.59	99.50%

**Table 3:** Overall classification accuracy of the three different structures.

Structure	FC6-SVM	FC7-SVM	FC8-SVM
Accuracy	99.19%	99.50%	94.87%
